# Supplementary material for: Development of two mouse strains conditionally expressing bright luciferases with distinct emission spectra as new tools for in vivo imaging
Source: Lab Anim (NY). 2023 Sep 7;52(10):247–57. doi: 10.1038/s41684-023-01238-6 (PMC10533401; doi:10.1038/s41684-023-01238-6)
Supplement: Supplementary file 2 — Reporting Summary [file 41684_2023_1238_MOESM2_ESM.pdf]

nature portfolio

Corresponding author(s): Toshiaki Nakashiba

Last updated by author(s): Jul 13, 2023

Reporting Summary

Nature Portfolio wishes to improve the reproducibility of the work that we publish. This form provides structure for consistency and transparency in reporting. For further information on Nature Portfolio policies, see our Editorial Policies and the Editorial Policy Checklist.

Statistics

For all statistical analyses, confirm that the following items are present in the figure legend, table legend, main text, or Methods section.

n/a	Confirmed	
☐	☒	The exact sample size (*n*) for each experimental group/condition, given as a discrete number and unit of measurement
☐	☒	A statement on whether measurements were taken from distinct samples or whether the same sample was measured repeatedly
☐	☒	The statistical test(s) used AND whether they are one- or two-sided<br>*Only common tests should be described solely by name; describe more complex techniques in the Methods section.*
☒	☐	A description of all covariates tested
☐	☒	A description of any assumptions or corrections, such as tests of normality and adjustment for multiple comparisons
☐	☒	A full description of the statistical parameters including central tendency (e.g. means) or other basic estimates (e.g. regression coefficient) AND variation (e.g. standard deviation) or associated estimates of uncertainty (e.g. confidence intervals)
☐	☒	For null hypothesis testing, the test statistic (e.g. *F*, *t*, *r*) with confidence intervals, effect sizes, degrees of freedom and *P* value noted<br>*Give P values as exact values whenever suitable.*
☒	☐	For Bayesian analysis, information on the choice of priors and Markov chain Monte Carlo settings
☒	☐	For hierarchical and complex designs, identification of the appropriate level for tests and full reporting of outcomes
☒	☐	Estimates of effect sizes (e.g. Cohen's *d*, Pearson's *r*), indicating how they were calculated

Our web collection on statistics for biologists contains articles on many of the points above.

Software and code

Policy information about availability of computer code

Data collection	BioTek Synergy HTX (Agilent Technologies, CA, USA) for tissue extract assays.<br>LumiFl-Spectrocapture AB-1850 instrument (Atto, Tokyo, Japan) for measurement of the emission spectra.<br>Nivo S (PerkinElmer, MA, USA) for measurement of the Km values.<br>VISQUE InVivo Smart-LF (Vieworks, Gyeonggi-do, Korea) for ex vivo imaging.<br>ImagEM 9100-13 (Hamamatsu Photonics, Shizuoka, Japan) for in vivo imaging.<br>Keyence GFP-lighting system (VB-L12, Keyence, Osaka, Japan) for GFP whole body imaging.<br>alpha-7SII digital color camera (Sony, Japan) for real time imaging of live mice.
Data analysis	CellSense software (Olympus,Tokyo, Japan), ImageJ software (version 1.53a, NIH) and CleVue (Vieworks, Gyeonggi-do, Korea) were used for data analysis.

For manuscripts utilizing custom algorithms or software that are central to the research but not yet described in published literature, software must be made available to editors and reviewers. We strongly encourage code deposition in a community repository (e.g. GitHub). See the Nature Portfolio guidelines for submitting code & software for further information.

Data

Policy information about availability of data

All manuscripts must include a data availability statement. This statement should provide the following information, where applicable:

- Accession codes, unique identifiers, or web links for publicly available datasets
- A description of any restrictions on data availability
- For clinical datasets or third party data, please ensure that the statement adheres to our policy

The data obtained from this study are available from the corresponding author upon reasonable request.

Human research participants

Policy information about studies involving human research participants and Sex and Gender in Research.

Reporting on sex and gender	No human research participants in this study.
Population characteristics	*Describe the covariate-relevant population characteristics of the human research participants (e.g. age, genotypic information, past and current diagnosis and treatment categories). If you filled out the behavioural & social sciences study design questions and have nothing to add here, write "See above."*
Recruitment	*Describe how participants were recruited. Outline any potential self-selection bias or other biases that may be present and how these are likely to impact results.*
Ethics oversight	*Identify the organization(s) that approved the study protocol.*

Note that full information on the approval of the study protocol must also be provided in the manuscript.

Field-specific reporting

Please select the one below that is the best fit for your research. If you are not sure, read the appropriate sections before making your selection.

☒ Life sciences ☐ Behavioural & social sciences ☐ Ecological, evolutionary & environmental sciences

For a reference copy of the document with all sections, see nature.com/documents/nr-reporting-summary-flat.pdf

Life sciences study design

All studies must disclose on these points even when the disclosure is negative.

Sample size	Statistical methods were not used a priori to determine the sample sizes. The sample sizes used in this study are similar to those widely used in this field.
Data exclusions	No data exclusions in this study.
Replication	All studies were repeated at least twice with similar results.
Randomization	No randomization was applied in this study.
Blinding	No blinding was applied in this study.

Reporting for specific materials, systems and methods

We require information from authors about some types of materials, experimental systems and methods used in many studies. Here, indicate whether each material, system or method listed is relevant to your study. If you are not sure if a list item applies to your research, read the appropriate section before selecting a response.

Materials & experimental systems

n/a	Involved in the study
☐	☒ Antibodies
☒	☐ Eukaryotic cell lines
☒	☐ Palaeontology and archaeology
☐	☒ Animals and other organisms
☒	☐ Clinical data
☒	☐ Dual use research of concern

Methods

n/a	Involved in the study
☒	☐ ChIP-seq
☒	☐ Flow cytometry
☒	☐ MRI-based neuroimaging

Antibodies

Antibodies used	rabbit anti-Luc2 (catlogu number: PM016, MBL, Tokyo, Japan)
Validation	According to the websites of MBL, this antibody has been validated by at least 3 literatures.

Animals and other research organisms

Policy information about studies involving animals; ARRIVE guidelines recommended for reporting animal research, and Sex and Gender in Research

Laboratory animals	C57BL/6J mice at 2-10 months old were used. For embryo imaging, BALB/c females and Jcl:ICR females were used.
Wild animals	No wild animals were used in this study.
Reporting on sex	Sex-based analysis was not conducted in this study, because the biodistribution of substrates and luciferase expression levels were assumed to be similar in both sexes.
Field-collected samples	No field-collected samples were used in this study.
Ethics oversight	All experimental protocols and husbandry for mice were approved by the Institutional Animal Care and Use Committee of RIKEN Tsukuba Branch and University of Tsukuba, and all mice were cared for and treated humanely in accordance with the Committee's guiding principles.

Note that full information on the approval of the study protocol must also be provided in the manuscript.
